# Controlled information integration and bayesian inference

**DOI:** 10.3389/fpsyg.2015.00070

**Published:** 2015-02-04

**Authors:** Peter Juslin

**Affiliations:** Department of Psychology, Uppsala UniversityUppsala, Sweden

**Keywords:** linear additive integration, probability reasoning, base-rate neglect, working memory capacity, Bayesian inference

One of the oldest hypotheses in cognitive psychology is that controlled information integration^1^ is a serial, capacity-constrained process that is delimited by our working memory resources, and this seems to be the most uncontroversial aspect also of present-day dual-systems theories (Evans, 2008). The process is typically conceived of as a sequential adjustment of an estimate of a criterion (e.g., a probability), in view of successive consideration of inputs to the judgment (i.e., cues or evidence). The “cognitive default” seems to be to consider each attended cue in isolation, taking its impact on the criterion into account by adjusting a previous estimate into a new estimate, until a stopping rule applies (e.g., Juslin et al., 2008).

Considering each input in isolation, without modifying the adjustments contingently on other inputs to the judgment, invites *additive integration*. The limits on working memory moreover contribute to an illusion of linearity. If people, when pondering the relationship between variables *X* and *Y*, are constrained by working memory to consider only two *X*–*Y* pairs, the function induced can take no other form than a line. As illustrated by many scientific models, with computational aids people can capture also non-additive and non-linear relations. But without support, this is rather taxing on working memory and additive integration, typically as a weighted average, seems to be the default process (Juslin et al., 2009), and, even more so, considering that additive integration is famously “robust” (Dawes, 1979), allowing little marginal benefit from also considering the putative configural effects of cues. These cognitive constraints therefore define a point toward which our judgments naturally gravitate.

This simplistic and probably not overly controversial model of controlled integration immediately has important consequences for our abilities to make judgments, some of which are well-known, some of which may still need to be further digested. At a general level, the most fundamental constraint on people's ability to comprehend and control their environment is this tendency to view it in terms of an “additive caricature,” as if they “looked at the world through a straw,” appreciating each factor in isolation, but with limited ability to capture the interactions and dynamics of the entire system. In more prosaic terms, a wealth of evidence suggests that multiple-cue judgments are typically well described by simple linear additive models (Brehmer, 1994; Karelaia and Hogarth, 2008), even if the task departs from linearity and additivity.

There are important exceptions where people transcend this imprisonment in a linear additive mental universe also without external computational aids, in particular, an ability to use a prior input to “contextualize” the meaning of an immediately following input. For example, for a lottery, like a 0.10 chance of winning $100 and $0 otherwise, people have little difficulty with contextualizing the outcome in view of the preceding probability; that is, to discount the “appeal” of the positive outcome of receiving $100 by the fact that the probability of ever seeing it is low. Likewise, people often have little difficulty with understanding normalized probability ratios and appreciate that, say, “30 chances in 100” and “300 chances in 1000” describe comparable states of uncertainty, something that again requires that one input is contextualized by another^2^. These exceptions are important, but seem to be connected to specific judgment domains.

## Controlled integration and probability theory

This contrasts with the requirements for multiplication implied by many rules of probability theory. We have therefore argued that additive combination may be an important—and often neglected—constraint on people's ability to reason with probability. Nilsson et al. (2009) proposed that even a classic bias, like the conjunction fallacy (Kahneman and Frederick, 2002), may not primarily be explained by specific heuristics *per se*, like “representativeness,” as typically claimed (although people sometimes use representativeness to make these judgments), but by a tendency to combine constituent probabilities by additive combination (see also Nilsson et al., 2013, 2014; Jenny et al., 2014). For example, people may appreciate that a description of “Linda” is likely if she is a feminist and unlikely if she is a bank teller (which might be mediated by “representativeness”), but knowing no feminist bank tellers they combine these assessments as best they can, which typically comes out as a weighted average (Nilsson et al., 2009). The rate of conjunction errors indeed seems equally high regardless of whether the representativeness heuristic is applicable or not (Gavanski and Roskos-Ewoldsen, 1991; Nilsson, 2008).

Juslin et al. (2011) similarly argued that base-rate neglect may be explained not by use of specific heuristics *per se*, but by additive combination of base-rates, hit-rates, and false alarm rates, where the weighting of the components is context-dependent (and more often neglect false-alarm rates than base-rates)^3^. Importantly, the reliance on additive integration is by no means arbitrary: to the extent that people base their judgments on noisy input (e.g., small samples), linear additive integration often yields as accurate judgments as reliance on probability theory, possibly explaining why the mind has evolved with little appreciation for the integration implied by probability theory (Juslin et al., 2009).

A strong example of problems with probability integration comes from studies of experienced bettors that have played on soccer games at least a couple of times each month for a period of 10 years or more (Nilsson and Andersson, 2010; Andersson and Nilsson, in press). They were extremely accurate in their translation of odds into probabilities, including that they aptly captured the profit margin introduced in the odds by the gambling companies. Yet, when they assessed the odds of an unlikely event *A* (i.e., an outcome of a soccer game), the odds for the conjunction of *A* and a likely event *B*, and the odds of the conjunction of *A*, *B*, and a third likely event *C*, their probability assessments and their willingness to pay for the bet, increased as likely events were added to the conjunction (the conjunction fallacy). This is predicted by a weighted average of the components, but violates probability theory. Exquisite assessment, but blatantly “irrational” integration, also in experienced and very motivated probability reasoners.

## Bayesian inference

Bayes' theorem in its odds format is,

(1) $$\begin{array}{l} p\left(H|E\right)/p\left(-H|E\right)\\ =p\left(H\right)/p\left(-H\right)\;\cdot p\left(E|H\right)/p\left(E|-H\right) \end{array}$$

where the left-hand side is the posterior odds for hypothesis *H* given evidence *E*, the first right-hand component is the prior odds for hypothesis *H*, and the second right-hand side is the likelihood ratio for the evidence *E*, given that *H* is true or false (i.e., −*H*). Equation (1) can be used to adjust your subjective probability that hypothesis *H* is true, in the light of evidence *E*.

Although apparently simple, the adjustment of the probability required in view of the evidence depends not only on the evidence attended at the moment, but on the prior probability (e.g., when the likelihood ratio is 2, you should adjust the prior probability of *H* upwards by 0.17 if the prior ratio is 1, but upwards by 0.04 if the prior ratio is 10)^4^. People do appreciate that the posterior probability is a positive function both of the prior and the evidence, but the impact of the prior is typically less than expected from Bayes' theorem (Koehler, 1996). If people, as argued above, are spontaneously inclined to adjust the probability of *H* (criterion) in the light of the new evidence *E* (the currently attended cue) independently of the previous input (captured in the prior probability), they will be affected by both priors and evidence, but not as much as with Equation (1), because they combine them additively^5^. This account explains why people find this a difficult task, but also suggests simplifying conditions and a “cure” for base-rate neglect.

A first example of a simplifying condition is natural frequencies (Gigerenzer and Hoffrage, 1995). If the base-rate problem immediately conveys the number of people with, say, a positive mammography test and the number of such people with breast cancer, people can “contextualize” the second number in terms of the first and directly appreciate that among positive tests, the proportion of breast cancer is low. In belief revision tasks, where the belief is repeatedly updated in the face of evidence, it has long been known that people successively average the “old” and “new” data (e.g., Shanteau, 1972; Lopes, 1985; Hogarth and Einhorn, 1992; McKenzie, 1994). An exception is when prior and evidence are presented in contextual and temporal contiguity, where people have some ability to “contextualize” their, presumably also here linear, weighting of the evidence in view of the prior, better emulating Bayesian integration (Shanteau, 1975).

The “cure” to base-rate neglect suggested by this view is, of course, to replace multiplicative integration with additive integration. An immediate implication is that people should have very little problem with certain kinds of “Bayesian updating;” for example, with updating their prior belief about the mean in a population after observing a new sample from the population. “Bayesian updating” here amounts to a (sample-size) weighted average between the “prior mean” and the “sample mean,” a task that people should be able to learn quite easily.

An example directly related to Bayes' theorem is provided in Juslin et al. (2011). In Experiment 1, each participant responded to 30 medical diagnosis tasks, in one of three formats: (i) *standard probability*, The base-rate, hit-rate, and false alarm rate were stated as probabilities^6^; (ii) *odds*, The same problem expressed in prior odds and likelihood ratios (Equation 1); (iii) *Log odds*, The same problems expressed as log odds, implying that one simply adds the log prior odds to the log likelihood odds to arrive at the log posterior odds. These are three ways to represent the same problems, but the first two formats require multiplication, the last one additive integration. Fifteen participants received *Metric instruction*, explaining and exemplifying the range and sign of the metric used, but with no guidance on how the integration should be made. The other 15, in addition, received *Computational instructions* on how to solve the problems, explaining how the components should be integrated according to Bayes' theorem with numerical examples.

**Figure 1:**
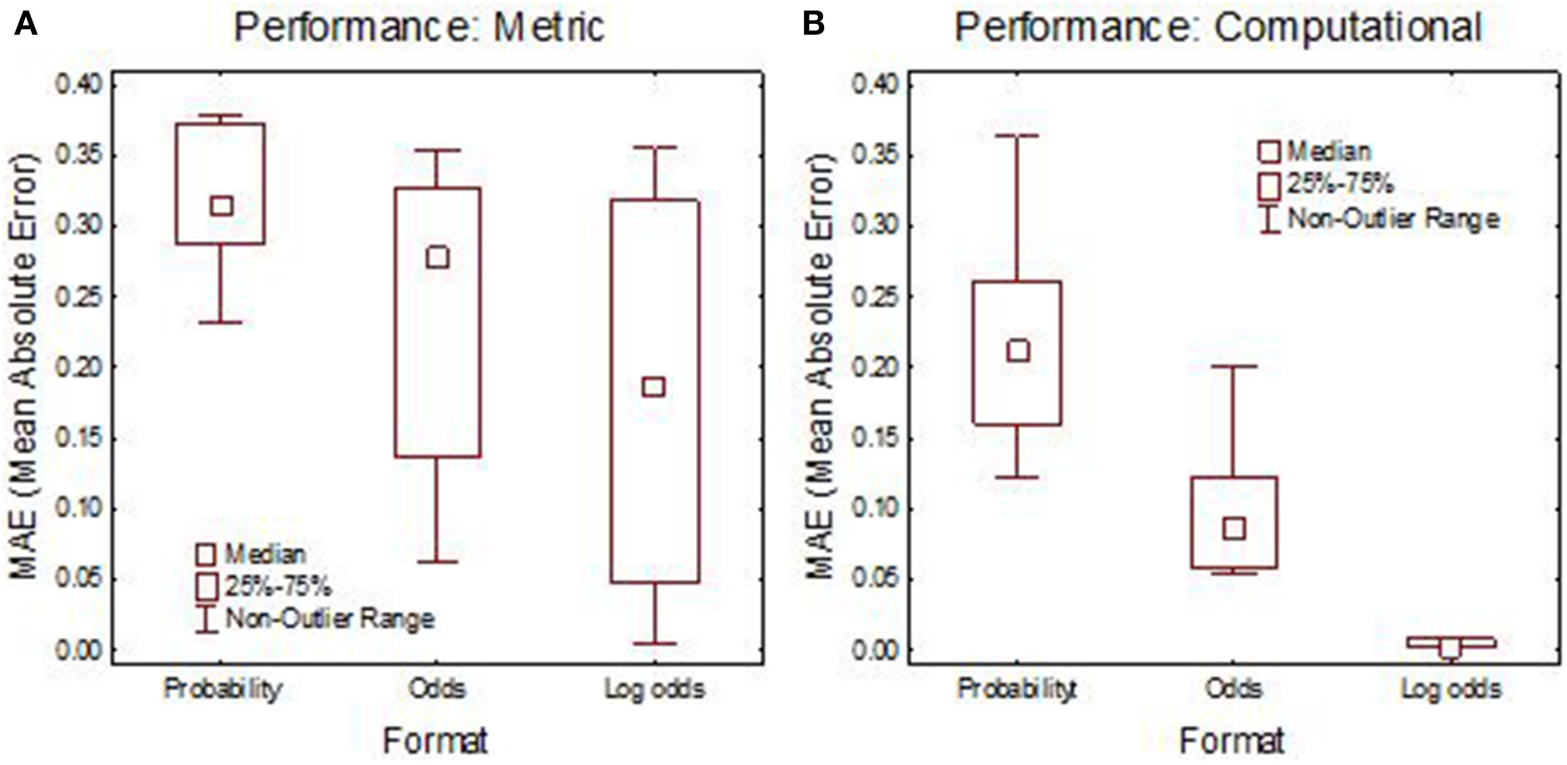
**Median performance in Experiment 1 in terms of Mean Absolute Error (MAE) between the judgment and Bayes' theorem. (A)** Metric instruction; **(B)** computational instruction. Adapted from Juslin et al. (2011) with permission.

The performance is summarized in Figure 1. Already with a Metric instruction, the log-odds format produced judgments closer to Bayes' theorem than the standard probability format. With computational instruction, the standard probability format produced poor performance and participants were still better described by an additive than a multiplicative (Bayesian) model. With log odds and computational instruction, performance was in perfect agreement with Bayes' theorem. People can thus flawlessly perform Bayesian calculation when the integration is additive, but when the format requires multiplication they are inept also after explicit instruction, still approximating Bayes' theorem as best they can by a linear additive combination.

## Conclusions

A caveat is that although these results demonstrate limits on *computational ability*, admittedly they do not address the important issue of *computational insight*: the understanding of what needs to be computed in the first place. Research has emphasized conditions that foster computational insight by highlighting subset relations that are important in Bayesian reasoning problems (e.g., Barbey and Sloman, 2007), perhaps at the neglect of the “old-school” information processing constraints on people's computational abilities discussed here. The “cure” suggested here is drastic in the sense that it requires people to think of uncertainty in an unfamiliar log odds format, and the extent to which they can learn to do this is an open question. The dilemma might well be that the probability format is more easily translated into action, because probabilities can be used directly to fraction-wise “contextualize” (discount) decision outcomes, but for reasoning about uncertainty people are better off with formats that allow additive integration.

### Conflict of interest statement

The author declares that the research was conducted in the absence of any commercial or financial relationships that could be construed as a potential conflict of interest.

## Footnotes

^1^Controlled processes refer to cognitive processes that are slow, conscious, intentional, and constrained by attention, in contrast to automatic processes that are rapid, not constrained by attention, and can be triggered also directly by stimulus properties (Schneider and Shiffrin, 1977; see also Evans, 2008). The claims about cognitive constraints discussed in this article refer to controlled processes and automatic processes may often better approximate Bayesian information integration (see, e.g., Tenenbaum et al., 2011).

^2^This ability is not perfect as illustrated by the phenomenon of denominator neglect (Reyna and Brainerd, 2008).

^3^A linear additive model captures many properties of the data, such that people do appreciate the qualitative effect of the base-rate, flexibly change their weighting as a function of contextual cues, and that the judgments are typically less extreme as compared to Bayes' theorem, but until we have a theory of how contextual cues affect the weight of the base-rate, we have limited ability to predict a priori how the base-rate will be used in a specific situation.

^4^With prior odds 1 and likelihood ratio 2, the posterior odds is 2 (Equation 1); an adjustment from a prior probability of 0.5 to a posterior probability of 0.67. With prior odds 10, the corresponding adjustment will be from 0.91 to 0.95.

^5^More specifically, when the base-rate is extreme, as in the “mammography problem” (e.g., Gigerenzer and Hoffrage, 1995) people will “underuse” the base-rate, but in problems with ambiguous base-rate, like in the urn problems studied by Edwards (1982), they will “overuse” the base-rate and thus appear “conservative.”

^6^Here is an example of a medical diagnosis task: The probability that a person randomly selected from the population of all Swedes has the disease is 2%. The probability of receiving a positive test result given that one has the disease is 96%. The probability of receiving a positive test result if one does not have the disease is 8%. What is the probability that a randomly selected person with a positive test result has the disease? Correct answer: 20%.
